# Effects of Tris(1,3-dichloro-2-propyl) Phosphate (TDCPP) in *Tetrahymena Thermophila*: Targeting the Ribosome

**DOI:** 10.1038/srep10562

**Published:** 2015-05-21

**Authors:** Jing Li, John P. Giesy, Liqin Yu, Guangyu Li, Chunsheng Liu

**Affiliations:** 1 College of Fisheries, Huazhong Agricultural University, Wuhan 430070, China; 2 Department of Veterinary Biomedical Sciences, and Toxicology Centre, University of Saskatchewan, Saskatoon, Saskatchewan, Canada S7N 5B3; 3 Department of Biology and Chemistry, City University of Hong Kong, Kowloon, Hong Kong, China; 4 School of Biological Sciences, University of Hong Kong, Hong Kong, SAR, China; 5 State Key Laboratory of Pollution Control and Resource Reuse, School of the Environment, Nanjing University, Nanjing, China

## Abstract

Tris(1,3-dichloro-2-propyl) phosphate (TDCPP) has been frequently detected in the environment, and exposure to TDCPP appears widespread. It has been implicated to cause toxicity in vertebrates, but its potential to affect lower-trophic-level species remains unknown. In the present study, the ciliated protozoan, *Tetrahymena thermophila*, was used as a model to evaluate toxic effects of TDCPP and explore molecular mechanisms by integrating phenotypic observation, RNA-Seq and transmission electron microscopy (TEM) Imaging technologies. Exposure to 0.01, 0.1 or 1 μM TDCPP for 5 days significantly decreased the relative biomass by reducing number of cells, size of cells and quantity of cilia in a dose-dependent manner. RNA-Seq analysis demonstrated that expression of twenty-one ribosome protein genes was down-regulated and these genes were enriched in “ribosome” term in KEGG pathway analysis. Furthermore, down-regulation of genes expressing ribosome proteins was accompanied by decreased ribosome quantity in rough endoplasmic reticulum and cytoplasm and enlarged ribosome size. Therefore, taken together, the data from the present study suggest that exposure to TDCPP affects growth and reproduction of *Tetrahymena thermophila* by targeting the ribosome. This information might provide insights into critical mechanisms of toxic action in other species and lead to useful bioindicators of exposure to TDCPP.

As the primary replacement of the phase-out retardant polybrominated diphenyl ether (PBDE), tris(1,3-dichloro-2-propyl) phosphate (TDCPP) has been increasingly used in various products, such as plastics, foams, textiles, varnishes, electronics equipment and furniture^1^. It has been reported that annual production of TDCPP in the United States is estimated to have been in the range of 4500 to 22,700 tons in 1998 and 2006^1^.

Recently, TDCPP has been reported to have been frequently detected in various environmental media, including indoor air, surface water, drinking water, influents, effluents, and exposure to the chemical in wildlife and human appears widespread^1^,^2^,^3^,^4^,^5^,^6^,^7^,^8^,^9^. For example, in China, TDCPP was detected in water samples from the Songhua River, with the concentrations of 2.5–40 ng/L^10^. In effluent of sewage treatment plants in Germany and Norway, TDCPP was detected with concentrations ranging from 20 ng/L to 740 ng/L^1^,^2^. Furthermore, TDCPP was detected in freshwater perch at 36–140 μg/kg lipid mass (lm)^5^. Recently, TDCPP and its metabolite (bis(1,3-dichloro-2-propyl) phosphate; BDCPP) were also detected in human milk and urine of office workers, which suggested that humans are being exposed^5^,^11^,^12^.

Despite its frequent detection in environmental media and occurrence of exposure of wildlife and humans, to date only limited information is available about the toxic effects and mechanisms of TDCPP. Exposure to TDCPP causes neurotoxicity, developmental toxicity, endocrine disruption and hepatotoxicity. For example, exposure to TDCPP promoted differentiation of neurons in PC12 cells and changed swimming behavior in larvae of zebrafish^13^,^14^,^15^; Early embryogenesis and larva development in chicken and zebrafish embryos are susceptible to effects of TDCPP, and molecular mechanisms included down-regulation of genes related to embryogenesis and up-regulation of proteins related to development of fast muscle and cartilage in zebrafish embryos^16^,^17^,^18^,^19^; Using cell lines and zebrafish as models, it was found that TDCPP could cause adverse effects on steroidogenesis, thyroid and nuclear-associated pathways, leading to endocrine disruption^20^,^21^,^22^,^23^,^24^,^25^,^26^; Recently, hepatotoxicity of TDCPP was observed in both zebrafish and chicken, evidenced by transcriptional alterations of related genes, occurrence of inflammation and histopathology in liver^19^,^27^,^28^.

Published data suggest that treatment with TDCPP causes neurotoxicity, developmental toxicity, endocrine disruption and hepatotoxicity in vertebrates, but to the best of our knowledge no information is available for evaluating effects of TDCPP in low-trophic-level species, such as *T. thermophila*. *T. thermophila* is a free-living ciliated protozoan with a global distribution in freshwater environments^29^. Recently, the genome of *T. thermophila* has been sequenced, and related molecular genetic technologies and genomic resources have been developed^30^,^31^,^32^,^33^,^34^. Due to its convenience for cultivation under laboratory conditions and sensitivity to chemical exposure, *T. thermophila* has been used as a model to evaluate effects of chemicals and explore molecular mechanisms for many years^35^,^36^,^37^,^38^,^39^,^40^,^41^,^42^,^43^. In this study, *T. thermophila* was used to evaluate effects of TDCPP. Specificlly, effects of TDCPP on morphology and molecular mechanisms using RNA-Seq technology were examined.

## Materials and Methods

### Cell Culture and Growth Curves

*T. thermophila* SB210 was obtained from the Institute of Hydrobiology, Chinese Academy of Sciences, Wuhan, China, and was cultured in super proteose peptone (SPP) medium (2% proteose peptone, 0.1% yeast extract, 0.003% Fe-EDTA, 100 units/mL penicillin G, 100 mg/L streptomycin sulfate, 0.025 mg/L amphotericin B, pH 7.0) at 30 °C with shaking at 135 rpm as described before^29^,^35^. In order to produce growth curves, cells that grew to mid-logarithmic phase were inoculated into new media (20 mL) in triangular flasks, with a final density of 2.72 × 10^4^ cells/mL, and were cultured at 30 °C with a shaking at 135 rpm. During culture period, relative biomass and cell density were measured every two hours between 10 and 26 h after seeding. Relative biomass was calculated by using OD values at 400 nm, and cell density was measured using haemocytometer after anaesthesia. Three biological replicates were included in this study.

### TDCPP Exposure Protocols

TDCPP was purchased from Sigma (St. Louis, MO, USA), and was dissolved in dimethyl sulfoxide (DMSO) as a stock solution. Experiments were conducted in two phases. According to the growth curves produced above, cell density was 4.23 × 10^5^ cells/mL at 12 h after seeding, and cells grew to platform period after 22 h. Therefore, for the first phase, cells were seeded at a density of 4.23 × 10^5^ cells/mL and exposed to 0, 1, 10, 100, 1000 or 10000 μM TDCPP for 8 h. After exposure, effects of exposures to TDCPP on biomass were determined. For the second phase, similar to the first part, cells were seeded at density of 4.23 × 10^5^ cells/mL and exposed to 0, 0.01, 0.1 or 1 μM TDCPP for 8 h. After that, cells in the control group were inoculated into new control media at a seeding density of 4.23 × 10^5^ cells/mL. For exposure groups, equal volume cells compared with control group were inoculated into new exposure media. The exposure was continued for another 8 h. The exposure process was repeated until a total exposure duration of 5-day. During the exposure period, biomass was monitored after 1-, 3- and 5-day exposure, and cell density, body size and cilia quantity was determined after 5-day exposure. There were three biological replicates for each concentration.

### RNA Isolation and Sequencing

In order to explore molecular mechanisms, cells exposed to the greatest concentration (1 μM) for 5 day were collected for transcriptomic analysis. There were three biological replicates for each concentration. RNA isolation and sequencing were performed by Novogene Bioinformatics Technology Co., Ltd (Beijing, China). Briefly, isolation of total RNA was performed using TRIzol reagent following the manufacturer’s instructions and then genomic DNA was removed using DNase I (Invitrogen, New Jersey, NJ, USA). RNA quality was determined using Agilent Bioanalyser 2100 (Agilent Technologies, Inc., Santa Clara, CA, USA), and concentration was measured using the ND-2000 (NanoDrop Technologies). mRNA was isolated using ligo-dT beads, and then fragmented in fragmentation buffer. Next, the short mRNA fragments were used as templates to synthesize first-strand cDNA with random hexamer primers, and then the second-strand cDNA was synthesized using dNTPs, DNA polymerase I and response buffer. The double-stranded cDNAs were purified using AMPure XP beads, and then used for end reparation, “A” base addition and finally were ligated with sequencing adapters. The adaptor-ligated fragments were size selected using AMPure XP beads. After quantification with Qubit 2.0 fluorometer (Life Technologies), cDNAs were used for PCR amplification and sequenced as 2 × 120 bp paired-end reads on Illumina HiSeq^TM^ 2000 sequencer (Illumina, San Diego, CA, USA).

### Sequence Tag Preprocessing and Mapping

Sequence tag preprocessing was performed according to a previously described protocol with some modifications^44^. Raw reads were cleaned by removing reads with adaptors, low quality (>50%) or high-proportion unknown bases (>10%) in a read. Clean data were mapped to *T. thermophila* Functional Genomics Database ( http://tfgd.ihb.ac.cn/) using Bowtie 2 software, with allowance of maximum 2 nucleotide mismatches.

### Gene Expression Calculation and Pathway Analysis

Gene expression was calculated using RPKM (reads per kilobase transcriptome per million mapped reads) method, with HTSeq software (union model). Differentially expressed genes were selected based on Padj value <0.05. KEGG (Kyoto Encyclopedia of Genes and Genomes) pathway analysis was performed using KOBAS (2.0) and corrected *P* value (FDR) cut-off was set at 0.05.

### Quantitative real-time polymerase chain reaction (RT-qPCR)

In order to confirm the findings of the RNA-Seq assay, a separate exposure experiment was conducted using the same exposure protocol as described above. *T. thermophila* were exposed to 0, 0.01, 0.1 or 1 μM TDCPP for 5 days, and then cells were collected for quantitative real-time PCR (RT-qPCR) and transmission electron microscopy imaging (only control and the greatest concentration). Isolation of total RNA, first-strand cDNA syntheses and RT-qPCR were performed using commercial kits as described previously^20^. Briefly, the isolation of total RNA was performed using TRIzol regent (Invitrogen, New Jersey, NJ, USA) following manufacturer’s instructions. RNA concentration and quality were assessed using the Epoch^TM^ Microplate Spectrophotometer (BioTek Instruments, Inc, Vermont, USA). After that, one microgram of total RNA was used for reverse transcription with Prime Script^TM^ RT reagent kit (Takara, Dalian, Liaoning, China). RT-qPCR was performed using SYBR^®^ Green Premix Ex Taq^TM^ II kits (Takara, Dalian, Liaoning, China), following manufacturer’s instructions, and melting curve was employed to check out purity and specificity of PCR productions in each assay. Sequences of primers were designed using Primer 3 software ( http://bioinfo.ut.ee/primer3-0.4.0/primer3/) (Table S1 in Supporting Information). According to the results of RNA-Seq and RT-qPCR, expression of adenosine/AMP deaminase family protein and cysteine proteinase 3 precursor stayed unchanged after TDCPP exposure, therefore they were used as internal control or housekeeping genes to which to normalize results to minimize variation between and among analyses. Thermal cycling was set at 95 °C for 10 min, followed by 40 cycles of 95 °C for 15 s and 58 °C for 1 min. RT-qPCR data were presented as fold change (log2) relative to control. There were three biological replicates for each concentration.

### Transmission Electron Microscopy (TEM) Imaging

After exposure, cells were collected and fixed in 2.5% glutaraldehyde. The samples were then sent to Wuhan Regional Centre of Life Science Instrument, Institute of Hydrobiology, Wuhan, China for TEM analysis. Briefly, cells were post-fixed using 1% osmium tetroxide for 3 h, and then were scraped, pelleted, dehydrated, infiltrated and embedded. After that, ultrathin sections were cut and stained with uranyl acetate. TEM images were taken using a Hitachi HT7700 TEM.

### Statistical Analyses

Statistical analyses of the data for the relative biomass, cell density, number of cilia, body size and gene expression (RT-qPCR) were performed using Kyplot Demo 3.0 software (Tokyo, Japan). Normality of data was evaluated using the Kolmogorov-Smirnow test and homogeneity was examined using Levene’s tests. If necessary, data were log-transformed to approximate normality. One-way analysis of variance was used to determine significant differences between the control and TDCPP exposure groups. A level of significance for type I error was set at *P* value <0.05.

## Results

### Growth Curves

**Figure 1:**
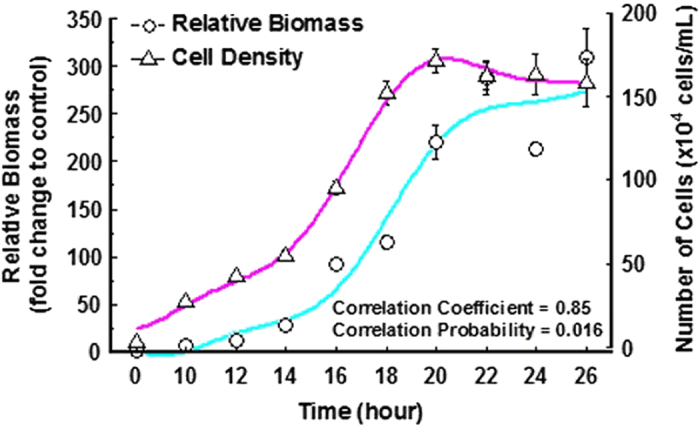
Growth curves based on relative biomass and cell density. Values represent mean ± SEM (n = 3). Spearman correlation analysis with Bonferroni correction was used to determine correlation coefficient and correlation probability. Curves were fitted using the Local Polynomial Regression method.

Growth curves of *T. thermophila* were produced by measuring time-dependent relative biomass and cell density between 10 and 26 h after seeding, and relative biomass and cell density reached platform stage between 20 and 22 h (Fig. 1). The correlation coefficient between the two curves was 0.85 (Fig. 1).

### TDCPP Decreases Biomass by Reducing Cell Density, Cell Size and Cilia Quantity

In the first part of exposure experiment, exposure to 100, 1000 or 10000 μM TDCPP for 8 h significantly decreased relative biomass, while no significant effect on biomass was observed after exposure to lower concentrations (1 or 10 μM) compared with the control (Figure S1, see Supporting Information). The calculated median effect concentration (EC_50_) was 615.1 μM (Figure S1, see Supporting Information).

**Figure 2:**
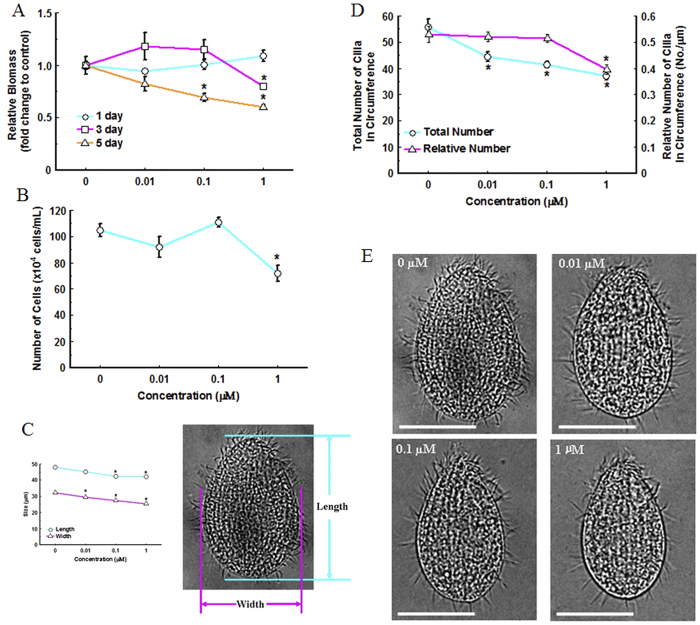
Dose-dependent effects on relative biomass (**A**), cell density (**B**), body size (**C**) and cilia quantity (**D**) and represented images (bar = 20 μM) (**E**) after exposure to 0, 0.01, 0.1 or 1 μM TDCPP for 5 days. Values represent mean ± SEM. Significant differences from the control are indicated by *P < 0.05. Each concentration contains 3 biological replicate.

In the second phase of the exposure experiment, no significant effect on biomass was observed after exposure to 0.01, 0.1 or 1 μM TDCPP for 1 days compared with the control. However, treatment with 1 μM TDCPP for 3 days, and 0.1 or 1 μM TDCPP for 5 days significantly decreased biomass (Fig. 2A).

Exposure to lower concentrations TDCPP (0.01, 0.1 or 1 μM) for 5 days significantly decreased the number of cells, size of cells or quantity of cilia (Figs. 2B-2E). Only exposure to the greatest concentration (1 μM) for 5 days significantly decreased number of cells by 28% compared with the control, while treatment with other concentrations (0.01 or 0.1 μM) did not affect numbers of cells (Fig. 2B). Mean length and width of *T. thermophila* in the control group were 47.98 and 32.14 μm, respectively. Exposure to 0.1 or 1 μM TDCPP for 5 days significantly reduced the mean length to 42.43 and 42.16 μm, respectively (Figs. 2C, 2E). Width was significantly less in all the exposure groups, with the width of 29.30, 27.35 and 25.28 μm in 0.01, 0.1 and 1 μM exposure group, respectively (Figs. 2C, 2E). Only exposure to the greatest concentration (1 μM) for 5 days significantly decreased relative number of cilia in circumference (cilia/mm circuference). However, the total number of cilia in circumference was significantly less in all exposure concentrations (Fig. 2D).

### Transcriptomic Responses to TDCPP

**Figure 3:**
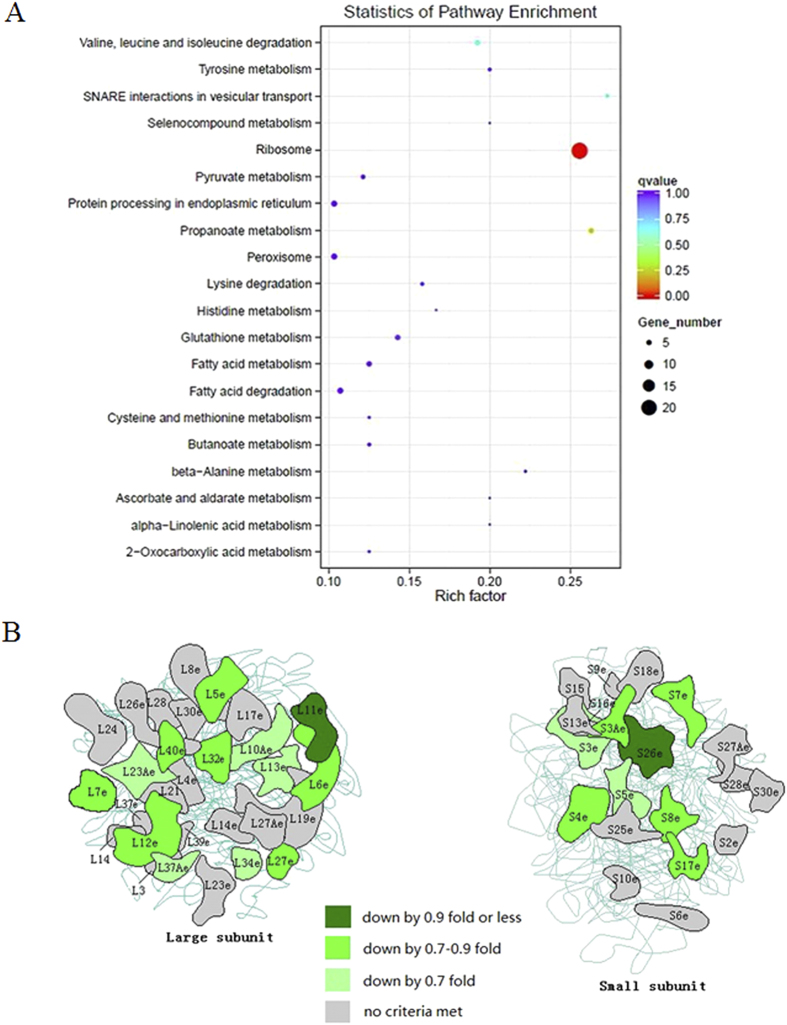
Significantly enriched KEGG terms (**A**) and expression profiles of genes involved in enriched “ribosome” term (**B**). Values represent mean ± SEM. Significant differences from the control are indicated by *P < 0.05. Each concentration contains 3 biological replicate. Figure 3B was modified from KEGG online figure ( http://www.genome.jp/kegg-bin/show_pathway?tet03010).

After filtering, about 50 million clean reads were obtained from each of the six libraries and over 92% of these reads were mapped to *T. thermophila* Functional Genomics Database ( http://tfgd.ihb.ac.cn/), representing a total of 25,606 genes. A total of 409 genes differentially expressed (72 up-regulated and 337 down-regulated) between control and TDCPP groups were identified using Padj value <0.05 as criteria. The most affected gene was ABC transporter family protein, which was significantly up-regulated by 7.18 fold (log2) compared with the control. When up- and down-regulated transcripts were further subjected to KEGG pathway analysis, the most significantly enriched pathway term was the ribosome (corrected *P* value = 1.08 × 10^−6^), where 21 down-regulated ribosome protein genes were included (Figs. 3A and B).

### RT-qPCR Validation

**Figure 4:**
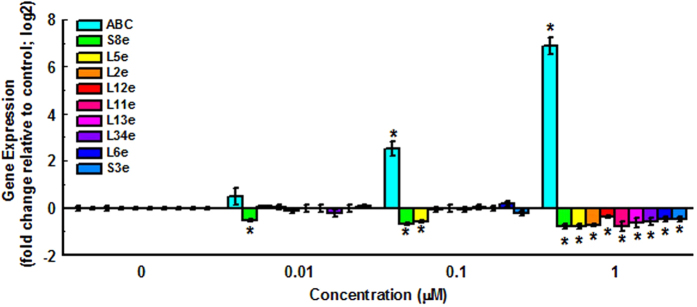
Dose-dependent expression profiles of genes randomly selected in enriched “ribosome” term. Values represent mean ± SEM. Significant differences from the control are indicated by *P < 0.05. Each concentration contains 3 biological replicate.

In order to confirm results observed during RNA-Seq analysis, a separate exposure experiment was conducted using the same exposure protocol, and four exposure concentrations (0, 0.01, 0.1 and 1 μM) were included. Ten genes were randomly selected to perform RT-qPCR assay. The results demonstrated that exposure to TDCPP caused a dose-dependent alteration in the expression of these genes, and expression profiles of the greatest concentration group were consistent with RNA-Seq data (Fig. 4).

### TDCPP Changes Ultrastructure of Cilia, Decreases Ribosome Quantity and Increases Ribosome Size

**Figure 5:**
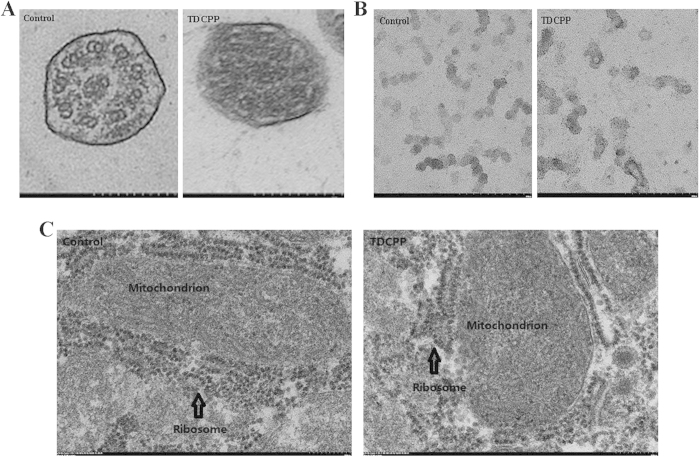
Represented transmission electron microscopy images for changed ultrastructure of cilia (**A**), decreased number of ribosome in rough endoplasmic reticulum (**B**) and cytoplasm (**C**) and enlarged ribosome in cytoplasm (**C**). bar = 200 nm.

To further confirm these findings, TEM was used to examine the effects of TDCPP on ultrastructure of cilia and number of ribosome and ribosome size. These results demonstrated that cilia had a typical “9+2” structure in control group, while the structure became unclear in 1-μM TDCPP group (Fig. 5A). Furthermore, exposure to 1-μM TDCPP for 5 days decreased the number of ribosome in rough endoplasmic reticulum and cytoplasm compared with the control, and ribosome size in cytoplasm was increased (Figs. 5B and 5C).

## Discussion

As primary replacement of the phased-out flame retardant PBDE, TDCPP has been increasingly used and environmental monitoring demonstrates that exposure to the chemical in wildlife and human appears widespread^1^,^2^,^3^,^4^,^5^,^6^,^7^,^8^,^9^. Toxic effects and molecular mechanisms of TDCPP have examined using vertebrates as models in previous studies^13^,^14^,^15^,^16^,^17^,^18^,^19^,^20^,^21^,^22^,^23^,^24^,^25^,^26^,^27^,^28^, but to the best of our knowledge no information is available for evaluating effects of TDCPP in low-trophic-level species. In this study, using *T. thermophila* as a model organism, toxic effects of TDCPP were examined and underlying molecular mechanisms were elucidated. Exposure to lesser concentration of TDCPP (0.01, 0.1 or 1 μM) for 5 days significantly decreased relative biomass, and the decrease of biomass was caused by toxic effects on growth and reproduction, evidenced by reduced number of cells, size of cells and quantity of cilia. Furthermore, molecular mechanisms seemed to involved down-regulation of ribosome protein genes revealed by RNA-Seq and KEGG pathway analysis and thus decrease of ribosome quantity and size enlargement of ribosome in rough endoplasmic reticulum and/or cytoplasm.

In order to evaluate effect of TDCPP on biomass, relative-biomass-calculation method was developed before performing exposure experiment. Previously, cell counter and luciferin-luciferase methods were usually used to measure effects of chemical on cell production in *T. thermophila*^29^,^38^, but the two methods can not be used to accurately measure biomass since they may cause biased results. For example, cell counter method was developed to quantify cells in certain volume of medium, thus it would ignore effect on other aspects associated to biomass, such as cell size. The luciferin-luciferase method were used detect total activities of cells in a well of plate, but it was based on measuring ATP content in cells and that content may be affected directly by chemical, thus leading to inaccurate measurements of biomass. In the present study, by using OD value at 400 nm to calculate relative biomass, we found that the relationship between two growth curves based on cell density and relative biomass was significantly correlated. Since this test was performed using the same batch cells without chemical exposure, quantity of cells was the only factor to affect biomass. Therefore, the results suggest that our method can be used to calculate relative biomass.

TDCPP exposure decreased relative biomass at lesser concentration by reducing growth and reproduction of *T. thermophila*. Biomass is defined as the total mass of living matter within a given unit of environmental area and associated with growth and reproduction of one species within certain period, representing a population-level parameter. In this study, using relative-biomass-calculation method developed, we found relative biomass was decreased only in great-concentration exposure groups (100, 1000 or 10000 μM) after 8-h exposure, but when lengthening exposure time to 5 days lesser-concentration exposure (0.1 or 1 μM) caused similar effect. Since *T. thermophila* has a short generation time (around 150 min), which allows a relatively large number of generations (about 10 generations) to be produced even during 24 h of exposure^45^,^46^, therefore, the results of present study suggest that TDCPP can cause toxic effects at lesser concentration by multi-generation exposure, thus might threaten wildlife upon exposure of environment-relevant concentration. Furthermore, biomass is associated to growth and reproduction statuses of one species within certain period, so we hypothesized that decrease in biomass in this study was caused by certain toxic effects of TDCPP on growth and reproduction. Therefore, we measured quantity of cells, size of cells and quantity of cilia in *T. thermophila* after TDCPP exposure. The results confirmed our hypothesis, evidenced by reduced quantity of cells, size of cells and quantity of cilia due to TDCPP exposure.

Molecular mechanisms involved in TDCPP-induced toxicity were revealed as down-regulation of ribosome protein genes and thus decrease of ribosome quantity, and enlargement of ribosome size. In order to explore molecular mechanisms, global gene expression was examined by using RNA-Seq technology, and we found that twenty-one ribosome protein genes with down-regulation were significantly enriched in “ribosome” term by KEGG pathway analysis. Ribosome is composed of ribosome protein and rRNA, and is responsible for protein synthesis in cell^47^. Therefore the down-regulation of ribosome protein genes would reduce production of corresponding ribosome proteins and thus reduce quantity of ribosome in cells, which was further supported by TEM images, evidenced by decreased quantity of ribosome in rough endoplasmic reticulum and cytoplasm. Furthermore, we observed enlargement of ribosome. It was calculated that enlargement of ribosome would change biological functions of ribosome, decreasing biogenesis of ribosome in cells. Previous study suggests that ribosome biogenesis drives cell growth and proliferation^48^, therefore down-regulation of ribosome protein genes may play a key role in TDCPP-induced phenotypic alterations, including decreased quantity of cells, reduced cell size and lessened cilia quantity. However, further studies are needed to elucidate mechanisms for down-regulation of ribosome protein genes and enlargement of ribosome size.

In summary, our data suggest that low-concentration TDCPP exposure decreases relative biomass of *T. thermophila* by reducing quantity of cells, size of cells and quantity of cilia, and molecular events seem to involved down-regulation of ribosome protein genes and thus the decrease in ribosome quantity and structural enlargement of ribosome, indicating ribosome as a main target in TDCPP-indcued toxicity. Therefore, our results for the first time provide evidences to support that TDCPP is toxic for *T. thermophila* at relative low concentrations and warrant the need for further studies to confirm these findings in other low-trophic-level species. In addition, mechanism information might provide insights into critical understandings of toxic action in other species and lead to useful bioindicators of exposure to TDCPP.

## Additional Information

**How to cite this article**: Li, J. *et al.* Effects of Tris(1,3-dichloro-2-propyl) Phosphate (TDCPP) in *Tetrahymena Thermophila*: Targeting the Ribosome. *Sci. Rep.* **5**, 10562; doi: 10.1038/srep10562 (2015).

## Supplementary Material

Supplementary Information

Supplementary Dataset 1
