# Supplementary material for: Effects of Tris(1,3-dichloro-2-propyl) Phosphate (TDCPP) in Tetrahymena Thermophila: Targeting the Ribosome
Source: Sci Rep. 2015 May 21;5:10562. doi: 10.1038/srep10562 (PMC4440212; doi:10.1038/srep10562)
Supplement: Supplementary Information [file srep10562-s1.doc]

**Supporting Information**

**Effects of Tris(1,3-dichloro-2-propyl) Phosphate (TDCPP) in *Tetrahymena Thermophila*: Targeting the Ribosome**

Jing Li^1^, John P. Giesy^2,3,4,5^, Liqin Yu^1^, Guangyu Li^1^,*, Chunsheng Liu^1^,*

^1^College of Fisheries, Huazhong Agricultural University, Wuhan 430070, China

^2^Department of Veterinary Biomedical Sciences, and Toxicology Centre, University of Saskatchewan, Saskatoon, Saskatchewan, Canada S7N 5B3

^3^Department of Biology and Chemistry, City University of Hong Kong, Kowloon, Hong Kong, China

^4^School of Biological Sciences, University of Hong Kong, Hong Kong, SAR, China

^5^State Key Laboratory of Pollution Control and Resource Reuse, School of the Environment, Nanjing University, Nanjing, China

***Corresponding authors:**

Guangyu Li (G. Li)

Chunsheng Liu (C Liu)

College of Fisheries

Huazhong Agricultural University

Wuhan 430070, China

Tel: 86 27 87282113

Fax: 86 27 87282114

Emails: [ligy2001@163.com](mailto:ligy2001@163.com) (Dr. Guangyu Li)

[liuchunshengidid@126.com](mailto:liuchunshengidid@126.com) (Dr. Chunsheng Liu)

Table S1. Sequences of primers for the genes tested.

| Gene Name | Gene ID | Sense primer（5’-3’） | Antisense primer （5’-3’） | Product length (bp) |
| --- | --- | --- | --- | --- |
| Adenosine/AMP deaminase family protein | [TTHERM_00895970](http://tfgd.ihb.ac.cn/search/detail/gene/TTHERM_00895970) | ATTGCCAAGAAGCATCATCC | TCACCACCATGGAAAACAAA | 161 |
| Cysteine proteinase 3 precursor, putative | TTHERM_00191270 | TTAGCTACTGCTGGCCCAAT | AATGCTCACCCCAACTGTTC | 187 |
| ABC transporter family protein | TTHERM_00035330 | ACCCAGAAACTCACGCAGAC | TGCATACATTTTGGCGTTTT | 164 |
| S3e | TTHERM_01151500 | CAGAATTAAGGCCACCAAGC | TAAGCAGAGGCGCATAAACC | 159 |
| S3Ae | TTHERM_00047480 | CCAAGAACCTCATCGGAGAA | ACCCTTCTTTTCGTGGGAGT | 161 |
| S4e | TTHERM_00149300 | TAACCCCGAAATCAAGATCG | GCATCCTTAACGTGGCAAAT | 189 |
| S5e | TTHERM_01386050 | GAAGGCTCTCTGCATCAAGG | TTTTCTGACGACACCAGCAG | 154 |
| S8e | TTHERM_01151600 | AAAGGGCAGACCCATCAGTA | TGTTTTCAGAACCCCAAGAGA | 155 |
| S7e | TTHERM_00471090 | ACACCCACCCTGATTTGAAG | TCGAGTTCGATGATGAGCTTT | 172 |
| S17e | TTHERM_00762890 | AGGGTCCCGTCAGAGGTATT | GGAAGCGCTAACAAGACCAG | 176 |
| S26e | TTHERM_00463490 | TATGGTCGATCCCTCTTCCA | TCTTCTGTCTTCAGCGCATC | 157 |
| L5e | TTHERM_00736480 | CAGAGTCCTCTGTGCTGCTG | CGACATTGAAGTAGGCACCA | 182 |
| L6e | TTHERM_00136120 | TCGGTAGAGAAAAGGCCAGA | AGGTGAACTTGGAGGCAAGA | 189 |
| L7e | TTHERM_00721150 | CTTTCGGTTATCCCACCAGA | CCAACAGTGGTGATTTCGTG | 172 |
| L10Ae | TTHERM_00577240 | GCTGGTATTGCTTGCATTGA | AACGACGGATTCACCTTCAG | 198 |
| L11e | TTHERM_00829380 | ATCAACTGTTGCGTTGGTGA | TGGTAACGTGAACAGCCATC | 157 |
| L12e | TTHERM_00028740 | TTGCAACCAACACAAGGGTA | TGAGGTCACCAGAGTGCTTG | 167 |
| L13e | TTHERM_00578640 | TTAATGACACCGCTGACACC | TCTTTCCATCCCACTTTTGG | 174 |
| L23Ae | TTHERM_00134940 | AGGTTGCCAAGAAGGGATCT | TTCATGGCCTTTTCAGTGGT | 193 |
| L27e | TTHERM_00780960 | TTCGCTGGTAAGAAGGCTGT | AAGGGCTTGACGGAAGTTCT | 161 |
| L32e | TTHERM_00085180 | TAAGATCGGTTTCGCCTCTG | CCGAGTTCAGCAGCTCTCTT | 189 |
| L34e | TTHERM_00149630 | GGCCCAAAGAATCACCTACA | GTGGCGTATTCAGCAGGTCT | 195 |
| L37Ae | TTHERM_00075670 | CCCTCAGAAAGGTCGTCAAG | AGGGGTGGTAAGTTCCCAAG | 161 |
| L40e | TTHERM_00339620 | TATCTTCGCCGGTAAGCAAT | GCAAACCTTCTTTTCGCAGT | 160 |

**Figure S1**


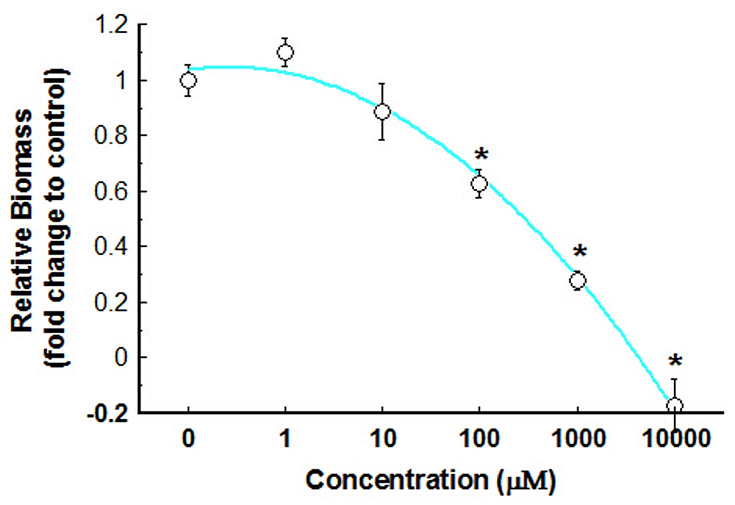


**Figure S1:** Dose-dependent effects on relative biomass after exposure to 0, 1, 10, 100, 1000 or 10000 µM TDCPP for 8 h. Values represent mean ± SEM. Significant differences from the control are indicated by **P* < 0.05. Each concentration contains 3 biological replicate. Curves were fitted using the Local Polynomial Regression method.
